# Damselfly eggs alter their development rate in the presence of an invasive alien cue but not a native predator cue

**DOI:** 10.1002/ece3.7729

**Published:** 2021-06-24

**Authors:** Andrzej Antoł, Szymon Sniegula

**Affiliations:** ^1^ Institute of Nature Conservation Polish Academy of Sciences Kraków Poland

**Keywords:** *Ischnura elegans*, invasion biology, Naïve prey hypothesis, signal crayfish, spinycheek crayfish

## Abstract

Biological invasions are a serious problem in natural ecosystems. Local species that are potential prey of invasive alien predators can be threatened by their inability to recognize invasive predator cues. Such an inability of prey to recognize the presence of the predator supports the naïve prey hypothesis. We exposed eggs of a damselfly, *Ischnura elegans*, to four treatments: water with no predator cue (control), water with a native predator cue (perch), water with an invasive alien predator cue (spinycheek crayfish) that is present in the damselfly sampling site, and water with an invasive alien predator cue (signal crayfish) that is absent in the damselfly sampling site but is expected to invade it. We measured egg development time, mortality between ovipositing and hatching, and hatching synchrony. Eggs took longer to develop in the signal crayfish group (however, in this group, we also observed high green algae growth), and there was a trend of shorter egg development time in the spinycheek crayfish group than in the control group. There was no difference in egg development time between the perch and the control group. Neither egg mortality nor hatching synchrony differed between groups. We suggest that egg response to signal crayfish could be a general stress reaction to an unfamiliar cue or an artifact due to algae development in this group. The egg response to the spinycheek crayfish cue could be caused by the predation of crayfish on damselfly eggs in nature. The lack of egg response to the perch cue could be caused by perch predation on damselfly larvae rather than on eggs. Such differences in egg responses to alternative predator cues can have important implications for understanding how this group of insects responds to biological invasions, starting from the egg stage.

## Graphical abstract

Damselfly eggs were exposed to three predators cues: 1 native—perch and two alien crayfish: signal (not present in sites where damselflies were collected) and spinycheek (present in site where damselflies were collected). Egg development time, hatching synchrony, and eggs mortality were checked. Eggs developed to hatching faster in the presence of spinycheek crayfish (egg predator), slower in signal crayfish group, and no effect of perch (larvae predator) were found.

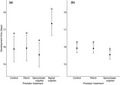

## 1 INTRODUCTION

Biological invasions are an increasing problem in many ecosystems worldwide (Lenda et al., 2019; Livingstone et al., 2020; Measey et al., 2020; Simberloff & Rejmánek, 2011). Invasive alien species (IAS) often reshape ecosystems by negatively affecting native communities through competition (Dickman, 2011; Gurevitch, 2011), predation (Clout & Russell, 2011), and habitat alteration (Boland, 2016). Additionally, IAS frequently cause economic problems through crop destruction and have impacts on forestry or fisheries (Davis, 2011). One of the ways in which the impact of invasive species on local communities increases is the lack of the ability of native species to recognize aliens as potential competitors or predators (Ferrari et al., 2015; Sih et al., 2010). Unrecognized competitors or predators can be an important component of a successful invasion by an alien species. Lack of recognition of IAS is especially important in predator–prey systems, with the predator being an IAS and the prey being a native species (the system can of course act reversely when an invasive prey is not consumed by a naïve native predator). The naïve prey hypothesis states that prey are more prone to be attacked by invasive alien predators because prey have not yet evolved sensing ability to detect these predators. If the naïve prey hypothesis is supported, it may explain why some IAS are so successful in settling in new habitats (Cox & Lima, 2006). Prey naïveté is more pronounced in aquatic ecosystems, where chemical sensing is more important than visual sensing (Anton et al., 2020; Cox & Lima, 2006).

Predators affect prey by direct consumptive effects, and the presence of predators per se can also impact prey. How and why these nonconsumptive predator effects impact prey are intriguing questions (Ferrari et al., 2010; Kobak & Kakareko, 2011; Sniegula, Nsanzimana, et al., 2019). The presence of chemical signals (cues) coming from a predator, called kairomones, in the environment can trigger a variety of prey responses. For example, kairomones directly affect prey feeding behavior (Naddafi et al., 2007), the predation efficiency of prey (Bucciarelli et al., 2018), passive defense traits (Czarnoleski et al., 2011), metabolic rate (Antoł et al., 2018), and life‐history traits (Czarnołęski et al., 2006; Sniegula, Nsanzimana, et al., 2019; Sniegula et al., 2020). However, we still do not have enough information on the effects of predator cues on prey traits during the initial developmental stage, that is, the egg stage (but see Ireland et al., 2007; Li & Jackson, 2005; Miner et al., 2010; Sniegula, Nsanzimana, et al., 2019; Sniegula et al., 2020), and we do not know whether the naïve prey hypothesis holds for the egg stage.

Eggs are unable to react actively to predation risk, that is, they cannot escape, hide, or defend against predators. In such a situation, one would expect changes in egg life‐history traits, such as changes in hatching time and egg mortality, in response to nonlethal and lethal nonconsumptive effects of predation stress (Warkentin, 2011). We know that exposure to environmental stressors (kairomones, population density, pollutants, etc.) in some developmental stages can affect the subsequent developmental stages, even if a particular stressor is absent during the subsequent stage (Stoks & Córdoba‐Aguilar, 2012)—so‐called carry‐over effects (Räsänen et al., 2002; Sniegula et al., 2017, 2020; Stoks & Córdoba‐Aguilar, 2012). The difference in hatching time within a growth season may cause differences in terms of resource availability or predator avoidance (Murillo‐Rincón et al., 2017; Sniegula, Golab, et al., 2019). If early hatching is advantageous in terms of ecological interactions, we refer to priority effects (Sniegula, Golab, et al., 2019). The priority effect at the ecological scale resembles an evolutionary mechanism where earlier colonists can monopolize new sites and significantly impact the shape of the community (De Meester et al., 2002). However, we must remember that early hatching is not always adaptive, for example, in situations when predators are less dangerous to larvae later in season (Ferrari et al., 2010; Sih & Moore, 1993). Hatching synchrony should be higher under predation stress, as it decreases the probability of a particular individual being hunted (swamping effect) (Hamilton, 1971). However, a mechanism that can decrease hatching synchrony is a bet‐hedging strategy (Philippi & Seger, 1989; Simons, 2011). To buffer the negative outcome of synchronous hatching in an unpredictable environment, hatching is spread out over time; however, there is some degree of individual risk connected with the decision of hatching date. Hence, it is important to study the effects of nonconsumptive predators on egg life‐history traits with the inclusion of possible hatching strategies.

To test the naïve prey hypothesis, we conducted an experiment on aquatic eggs of a damselfly native to central Europe, *Ischnura elegans*. We expect a slower egg development rate, higher mortality, and increased hatching synchrony under stress conditions imposed by native predator cues and an absence of egg responses in these traits under stress conditions imposed by invasive alien predator cues. Our hypothesis is based on the fact that predator cues originating from native predators can affect hatching characteristics (Fontana‐Bria et al., 2017; Sniegula, Nsanzimana, et al., 2019). Slower egg development and increased egg mortality may be caused simply by physiological responses to stress imposed by native but not alien predators. Slower egg development can, however, be an adaptive response caused by changes in predator biology over time. In such a scenario, slower egg development may lead to delayed hatching when predator density is reduced or when predators reach larger sizes and switch to alternative prey (actual for gap‐limited predators); or, as stated by Ferrari et al. (2010), delayed hatching may prolong susceptibility to predation during the egg stage. In the presence of native predators, we predict more synchronized hatching due to the swamping effect. However, other mechanisms such as bet hedging can impose pressure that tends to lower synchrony in general.

## 2 MATERIALS AND METHODS

### 2.1 Animal collection and maintenance

*Ischnura elegans* is a common damselfly species in Europe. Adult damselflies lay eggs into partly decomposed water plant tissues, and eggs and larvae develop in aquatic environments until emergence (Corbet, 1999). Damselfly eggs and larvae are prey for aquatic invertebrate and vertebrate predators (Corbet, 1999). We used three predators to impose nonconsumptive effects on damselfly eggs: perch (*Perca fluviatilis*), which is native and co‐occurs with *I. elegans* eggs and larvae; spinycheek crayfish (*Orconectes limosus*), an invasive alien predator that has co‐occurred with the damselfly for at least 50 years (Bonk & Bobrek, 2020; Śmietana, 2011); and signal crayfish (*Pacifastacus leniusculus*), an invasive alien predator that has not occurred in areas where we collected damselflies (Dobrzycka‐Krahel et al., 2017). Signal crayfish are expected to invade the damselfly sampling site in the near future; in 2020, new sites were found ca. 200 km from where the damselflies have been collected (M. Bonk and R. Maciaszek, personal communication). Perch predates mainly on damselfly larvae, while crayfish may also predate on eggs as crayfish forage in egg depositing sites (Hirsch et al., 2016). During the experiment, we noted damselfly egg development time and mortality between egg laying and hatching. Based on egg development time, we estimated hatching synchrony.

We collected adult *I. elegans* females by hand on June 13, 2020, from Bonarka Pond in Kraków (50°01′27.3″N 19°57′04.0″E). We collected only tandems to increase the probability that females were fertilized. Males were released. We transported females to the Institute of Nature Conservation Polish Academy of Sciences in Kraków (INC PAS) by car in individual plastic containers containing wet filter paper. The containers were placed in bigger styrofoam cooler equipped with a frozen cartridge to protect animals from overheating. The collection site was ca. 5 km from the INC PAS. We kept females in a room with an unregulated temperature and a natural photoperiod until they laid eggs, which occurred within four days after females had been collected in the field. These females were kept individually in 200‐ml (height – 9 cm, diameter – 4 cm) plastic cups with wet filter paper attached to the side for egg laying. Initially, we collected 21 females. These females were not fed when kept in the cups.

We collected perch from Dobczyce Lake in southern Poland (49°52′18.316′′N, 20°2′30.937′′E), spinycheek crayfish from Kryspinów Lake in southern Poland (50°3′0.461′′N, 19°47′20.85′′E), and signal crayfish from Hańcza Lake in northern Poland (54°15′9.522′′N, 22°48′36.86′′E). Signal crayfish were transported to the INC PAS by car in aerated travel containers and spinycheek crayfish in no aerated buckets due to very close distance for transport. The distances from the collection sites to the INC PAS were 32 km (perch), 15 km (spinycheek crayfish), and 600 km (signal crayfish). In the laboratory, the predators were separated by species and kept in aquaria with 52 L of dechlorinated and aerated tap water in the same cabinet with a constant temperature of 20°C regulated by an air conditioner facility. The densities of predators in aquaria were based on the basal metabolic rate equations obtained for perch from Enders et al. (2006) and for crayfish from Wheatly (1989). To keep total metabolic rates balanced, the biomass of crayfish had to be two times higher than the biomass of fish. After weighing, we kept two specimens of signal crayfish (wet mass 100 g), five specimens of spinycheek crayfish (wet mass 90 g), and two specimens of perch (wet mass 41.5 g) per experimental aquarium. We fed all predators frozen Chironomidae larvae (IT‐IchtyoTrophic, Stare Polichno, Poland) every second day (the larvae were thawed prior to feeding) and earthworms (MyWorms, Słupsk, Poland) once a week. Once a week, we changed 10 L of water in the predator aquaria. The predators had been housed in the laboratory conditions for at least one week before we started the experiment. Perch were collected and housed with permission from the Local Ethical Committee (ref. 394/2020). Crayfish were collected with permission from the Regional Directorate for Environmental Protection in Białystok (ref. WPN.6205.21.2020.ML) and Nature Reserve Hańcza Lake and housed with permission from the Regional Directorate for Environmental Protection in Kraków (ref. OP‐I.672.8.2020.MK1).

### 2.2 Egg laying and hatching

Twice a day (morning and afternoon), we checked for newly deposited damselfly eggs. If we confirmed egg deposition, we cut fragments of filter paper containing 20 eggs and placed each fragment with eggs in a separate 200‐ml plastic cup (*h* – 9 cm, *d* – 4 cm). Every cup was filled with 67 ml of dechlorinated tap water and 33 ml of water with and without predator cues. To introduce the predator cues, we used water from predator aquaria. As a control, we used dechlorinated tap water kept in the same type of aquarium as the aquaria with predators. From each female, we took four fragments of filter paper, each fragment containing 20 eggs, and assigned each fragment to a different predator cue, that is, control, perch, spinycheek crayfish, and signal crayfish. In summary, from each female, we used 80 eggs distributed between four treatment groups. In total, we obtained eggs from 9 females (replicate number), from which one female deposited enough eggs for only three treatment groups. For this female, we used her eggs for all treatment groups except for the control group. One female was excluded from analysis due to extremely low hatching success of her eggs. We placed cups with eggs in a thermostatic cabinet (ST700, Pol‐Eko Aparatura, Wodzisław Śląski, Poland) with a constant temperature of 22°C and a photoperiod of L:D16:8 hr. The cups were randomly distributed to the treatments. We replaced 33 ml of water in cups with water from the appropriate predator and control aquarium every second day. The half‐life of predator cues is between 0.2 and 126 hr (Van Buskirk et al., 2014), and a period of ca. 48 hr was a moderate value. After 13 days (based on our a priori experience that the minimal egg development time at 22°C was 14 days), we started checking for the presence of newly hatched larvae, and we checked this twice a day (09:00 and 21:00 local time). When we noticed newly hatched larvae, we removed them from the cup and counted the development time between egg laying and hatching to the nearest 0.5 days. Six days after the eggs had started to hatch, we counted all eggs that remained in the cups to confirm the initial number of eggs set in every cup. By counting the remaining eggs, we received information on the number of eggs from which larvae did not hatch. We counted the remaining eggs six days after hatching had started because egg hatching happens within this time for this damselfly (Sniegula, Nsanzimana, et al., 2019; Sniegula et al., 2020).

### 2.3 Statistical analysis

The data on egg development time (calculated for each hatched individual) were analyzed with a general linear mixed model with lme4 (Bates et al., 2015), lmerTest (Kuznetsova, 2017) for model fitting, the car (Fox & Weisberg, 2019) package for calculating *p* values of particular factors (Wald III Type *χ* ^2^ test), multcomp (Hothorn et al., 2008) for post hoc analysis, and ggplot 2 (Wickham, 2016) for graphics. Developmental time was a response variable. Treatment (four levels: perch, signal crayfish, spinycheek crayfish, and control) was an explanatory variable. Cup and female were random factors. Using residual plots, we visually judged the homogeneity of variance and normality of residuals.

We tested the data on egg survival with a generalized linear mixed model with binomial error distribution and logit link function using the same packages as those we used for development time analysis. The response variable was hatching success or failure. The explanatory variable was experimental treatment. Cup and female were random factors. Using residual plots, we visually judged the homogeneity of variance and normality of residuals. To test for hatching synchrony, we calculated a coefficient of variance of egg development time per cup and analyzed it with a general linear mixed model with the coefficient of variance as the response variable, experimental treatment as the explanatory factor, and female ID as the random factor. The assumptions of homogeneity of variance and normality of error distribution were judged as in development time analysis. If we found a significant treatment effect, we ran Tukey's HSD test for post hoc comparison.

In cups from the signal crayfish treatment group, we observed the growth of green algae from the common genus *Scenedesmus* (E. Wilk‐Woźniak personal communication). The algae grew on cup bottoms and filter papers with eggs, and their visually judged amount was apparently higher than that in other experimental treatment groups. Taking into account the possibility that algae could bias the experimental outcome by a direct effect of algae on hatching, we ran analyses with and without the signal crayfish group.

## 3 RESULTS

**FIGURE 1:**
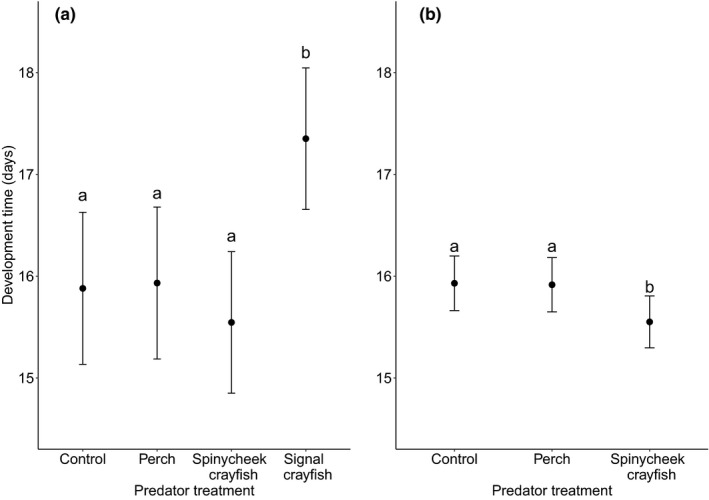
Least square means of egg development time between egg laying and hatching in *I. elegans*. Different letters denote significant differences at the 0.05 level. The differences between control, perch, and spinycheek crayfish group were not detectable when signal crayfish group was included in the analysis (a). When signal crayfish group was excluded from the model, eggs in spinycheek crayfish group developed faster (b)

Treatment significantly affected egg development time (*χ* ^2^ = 19.05, *p* < .001). Egg development time in the signal crayfish group was longer than that in the other groups (in all pairwise comparisons, *p* ≤ .01). After removing the signal crayfish group, the group effect was significant (*χ* ^2^ = 11.75, *p* = .003, Figure 1b). Eggs from the spinycheek crayfish group developed within a shorter time than did eggs from the perch (*p* = .01) and control (*p* = .008) groups. This result suggests that perch and invasive alien crayfish predator cues affect the hatching date of *I. elegans* in a nonuniform way.

**FIGURE 2:**
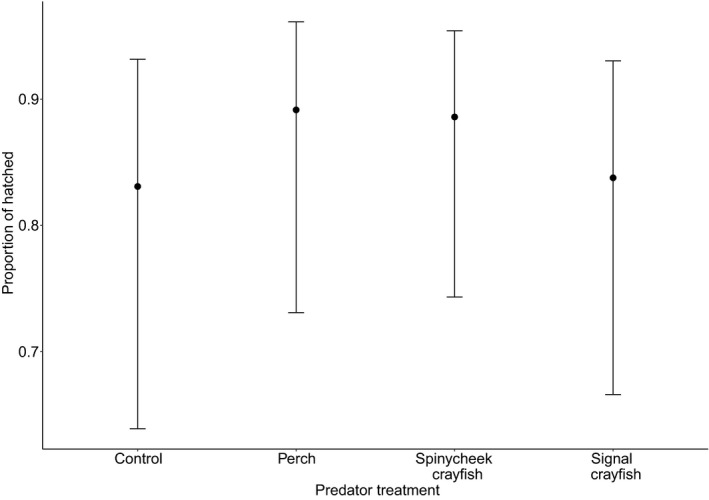
Proportion of hatched eggs of *I. elegans* grown in four predator treatments. There was no significant effect of predatory treatment

There was no group effect on egg survival (*χ* ^2^ = 1.02, *p* = .8, Figure 2). The result for egg survival did not change qualitatively when the signal crayfish group was removed from the analysis (*χ* ^2^ = 0.79, *p* = .67). This result suggests that perch and invasive alien crayfish predator cues do not affect egg survival of *I. elegans*.

**FIGURE 3:**
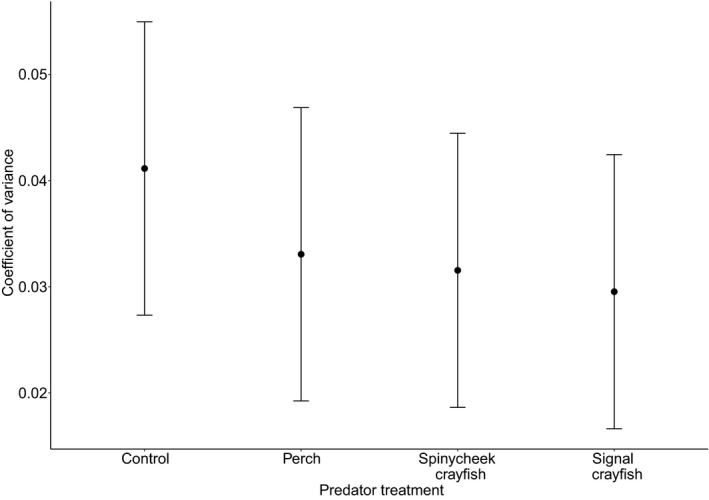
Least square means of the coefficient of variance in egg development time in *I. elegans* grown in four predator treatments. There was no significant effect of predator treatment

There was a nonsignificant group effect on hatching synchrony (*χ* ^2^ = 2.14, *p* = .54, Figure 3). The group effect was nonsignificant when the signal crayfish group was removed from the analysis (*χ* ^2^ = 0.94, *p* = .62). This result suggests that perch and invasive alien crayfish predator cues do not affect hatching synchrony in *I. elegans*.

## 4 DISCUSSION

We found that nonconsumptive effects of predator cues originating from different predators had different effects on egg development time until hatching in *I. elegans*. In particular, we observed no effect in the perch group, a trend for a shorter egg development time in the spinycheek crayfish group and a longer egg development time in the signal crayfish group. We noted that the egg response in the signal crayfish group might be confounded by green algae growth. These results do not support the naïve prey hypothesis. Furthermore, and contrary to our second and third predictions, predator cues did not alter the egg survival rate or hatching synchrony. The effect of predation cues on egg traits has previously been demonstrated in a number of experiments performed on aquatic organisms, such as the amphibians *Hyla regilla* and *Rana cascadae*, where predator cues led to earlier hatching (Chivers et al., 2001) and smaller hatchlings in the case of *Rana calamitans* (Ireland et al., 2007), a marine snail, *Nucella lamellosa* where predators decreased hatching success (Miner et al., 2010), and damselflies, where depending on the species, predator cues either accelerated (*Coenagrion pulchellum*) or decelerated (*Enallagma cyathigerum*) egg development rates until hatching (Sniegula, Nsanzimana, et al., 2019). However, current results are important and novel since they indicate that egg traits linked to fitness respond to native and IAS predator cues in a different and unexpected way.

Based on a meta‐analysis, Anton et al. (2020) report that on average, 200 generations are necessary for prey to recognize new predators. The studied damselflies and invasive alien spinycheek crayfish have co‐occurred in the sampling area through the last 50 years (Bonk & Bobrek, 2020; Śmietana, 2011). Assuming a maximum of two generations per year, *I. elegans* is still far from 200 generations. However, Anton et al. (2020) stated that previous experience with related species facilitates prey adaptation to cue recognition. The areas of the city of Kraków that are now colonized by spinycheek crayfish were inhabited by noble crayfish (*Astacus astacus*) and Danube crayfish (*Astacus leptodactylus*) (the Danube crayfish is also not native in the sampling region). The last records of noble crayfish in Kraków are from 2015 (Stanek et al., 2015), but the crayfish species was already extremely rare by then. Danube crayfish was observed in close vicinity of our damselfly collection pond in 2019 (M. Bonk personal communication). Hence, damselflies might associate IAS crayfish cues with native crayfish cues. Another explanation of the observed effect is that, in general, crustacean predators are not effective drivers of prey naïveté (Anton et al., 2020; Burraco & Gomez‐Mestre, 2016). Effective cue recognition could explain the altered *I. elegans* egg development time in the presence of spinycheek crayfish cues; however, the opposite was true during the experiment. In such scenarios, we would expect responses of *I. elegans* eggs to both spinycheek crayfish and native perch.

However, we did not observe an egg response to perch cues. We explain this result by perch‐specific predatory behavior. Damselflies lay eggs into partially or fully submerged rotting aquatic plant tissues, which are often close to the shore. Perch is very unlikely to forage there because such sites are rather inaccessible to predatory fish. The absence of a perch cue response in our study is supported by previous results from experiments on the nonconsumptive perch effect on damselfly eggs (Sniegula, Nsanzimana, et al., 2019). Despite this, the presence of perch cues during the egg stage decreased the larval growth rate (Sniegula et al., 2020). Interestingly, such a negative effect of fish cues on the larval growth rate occurred even if fish cues were present during the egg stage but absent in the larval stage, that is, the carry‐over effect (Sniegula et al., 2020; Stoks & Córdoba‐Aguilar, 2012). The presence of a carry‐over effect suggests that perch cues detected during the egg stage—a developmental stage during which perch is not dangerous to damselflies—can affect the following developmental stage when perch becomes a threat. The difference in reaction to perch cue can also be the result of some chemical characteristics of fish cue, but this needs further study.

The response of damselfly eggs to spinycheek crayfish cues is intriguing because we expected eggs to delay hatching when exposed to predator cues, given that the eggs recognize invasive crayfish cues as native crayfish cues. Crayfish can predate on damselfly eggs, as damselfly egg‐laying sites are available to crayfish (Hirsch et al., 2016). Crayfish may take advantage of this by direct predation on invertebrate eggs or eating them accidentally when foraging on rotting plant tissues. Therefore, accelerated egg development in the presence of crayfish cues may be an adaptation to escape the danger of predation. The same effect was present when *I. elegans* eggs were exposed to a mixture of conspecific and heterospecific damselfly larval cues (Fontana‐Bria et al., 2017). These results support our reasoning that egg predators may accelerate egg development time, while larval predators have no effect on egg development time.

We found that in the presence of signal crayfish cues, eggs took a longer time to develop. In this treatment, we observed green algae growth; thus, we treated these results with caution. Algal growth may affect egg development by limiting daylight access or creating hyperoxic conditions. Inhibition of development and growth by allelopathic effect of algal metabolites is also possible (Leflaive & Ten‐Hage, 2007). Hyperoxia could prolong egg development time, as has occurred in eggs of rainbow trout (Latham & Just, 1989). Green algae can also create hypoxia at night by consuming oxygen. However, during the experiment, we used a summer photoperiod, with the dark phase being shorter than the light phase. Therefore, intensive production and (in consequence) hyperoxia were more likely to occur. The allelopathic effect of algae on larval growth is also likely to occur. It was reported that *Scenedesmus acutus* extract inhibited growth of a moth *Spodoptera litoralis*; however, the effect was mediated by medium in which algae extract was diluted (Sharaby et al., 1993). Another possibility is that algae did not affect the eggs, and we observed an actual predator effect. Signal crayfish are absent in the damselfly collection site, and the closest area where the signal crayfish was very recently recorded is approximately 200 km away (Maciej Bonk, Rafał Maciaszek, personal communication). Older, stable populations of the signal crayfish are situated ca. 400 km from the damselfly collection site (Dobrzycka‐Krahel et al., 2017). It is therefore surprising that eggs developed at a slower rate in the presence of signal crayfish cues. One explanation could be that the signal crayfish cue is recognized by eggs as a potential danger without specification (Hawkins et al., 2004; Steinberg, 2012). Such an unspecified effect was demonstrated on predator avoidance behavior in the gammarids *Dikerogammarus villosus* and *Pontogammarus robustoides*. Here, gammarids reacted similarly to hungry fish of both sympatric (*Babka gymnotrachelus*) and allopatric (*Pygocentrus nattereri*) species (Jermacz et al., 2017). Finally, geographic dispersal of adult *I. elegans*, which is evident in damselflies (Johansson et al., 2013; Wellenreuther et al., 2011), could have permitted gene flow between populations having and not having contact with signal crayfish. However, this hypothesis needs confirmation.

Interestingly, we found no effects of predator cues on egg mortality. In contrast, previous results demonstrated a negative effect of native predator cues on egg survival in several damselfly species, including *I. elegans* (Sniegula, Nsanzimana, et al., 2019), and in marine snails (Miner et al., 2010). We suggest that the discrepancy may be caused by species‐ and population‐specific responses to predation cues. A previous experiment on *I. elegans* eggs was based on damselflies that originated from different populations (Sniegula, Nsanzimana, et al., 2019).

We expected increased hatching synchrony under predation danger due to the advantages of massive hatching, that is, the swamping effect. In such situations, a particular individual is statistically less prone to predator attacks (Hamilton, 1971). The lack of differences in hatching synchrony between treatments could be caused by a high synchrony of hatching in *I. elegans* (Corbet, 1999). Possible differences in hatching synchrony could occur at resolutions lower than 12 hr, but this question requires further investigation. We observed generally high synchrony in hatching time; therefore, we did not see the possible presence of a bet‐hedging strategy in the studied species.

In summary, we show that native and IAS predator cues alter egg life‐history responses in *I. elegans* with different strengths and directions. These differences in egg responses to alternative predator cues can have important implications for understanding how this group of insects responds to biological invasions, starting from the initial developmental stage. Future studies should focus on the mechanism of egg responses to alternative predators. For example, measuring changes in egg physiological parameters in response to the presence/absence of predator signals, as well as the carry‐over effects of predator cues measured during larval and adult stages, would provide further insights into predator–prey interactions.

## CONFLICT OF INTERESTS

The authors declare no conflicting interests.

## AUTHOR CONTRIBUTIONS

**Andrzej Antoł:** Conceptualization (equal); Data curation (lead); Formal analysis (lead); Investigation (lead); Methodology (equal); Project administration (equal); Resources (equal); Software (lead); Validation (equal); Visualization (lead); Writing‐original draft (lead); Writing‐review & editing (equal). **Szymon Sniegula:** Conceptualization (equal); Funding acquisition (lead); Investigation (supporting); Project administration (equal); Supervision (supporting); Validation (equal); Writing‐review & editing (equal).

## Data Availability

All data used in this manuscript are available at this link: https://figshare.com/articles/dataset/Damselflies_eggs_development/13241483.
